# A one-dimensional parameter-free model for carcinogenesis in gene expression space

**DOI:** 10.1038/s41598-022-08502-8

**Published:** 2022-03-19

**Authors:** Roberto Herrero, Dario A. Leon, Augusto Gonzalez

**Affiliations:** 1 Institute of Metrology, 10200 Havana, Cuba; 2 Institute of Cybernetics, Mathematics and Physics, 10400 Havana, Cuba; 3 S3 Centre, Istituto Nanoscienze, CNR, 41125 Modena, Italy

**Keywords:** Power law, Gene expression, Cancer models

## Abstract

A small portion of a tissue defines a microstate in gene expression space. Mutations, epigenetic events or external factors cause microstate displacements which are modeled by combining small independent gene expression variations and large Levy jumps, resulting from the collective variations of a set of genes. The risk of cancer in a tissue is estimated as the microstate probability to transit from the normal to the tumor region in gene expression space. The formula coming from the contribution of large Levy jumps seems to provide a qualitatively correct description of the lifetime risk of cancer in 8 tissues, and reveals an interesting connection between the risk and the way the tissue is protected against infections.

## Introduction

Cancer is a complex multifactorial phenomenon, the understanding of which is still a challenge. The current knowledge of carcinogenesis emphasizes on a sequence of special (driver) mutations leading to a progression to the tumor state. Epigenetic changes, microenvironment effects and other factors are also recognized to play important roles^1^. There is also a plausible hypothesis that cancer is a remnant of an ancient multicellular state encoded in our genes^2^.

Existing theories face difficulties and should make additional assumptions. Let us examine, for example, the prototype of multistep theory: Vogelstein’s idea of progression in colon cancer^3^. In order to implement it in an algorithm, we should introduce as additional parameters the number of intermediate steps and their transition rates.

In the present paper, we advance a model of tumorigenesis in which parameters are either calculated from processed gene expression data or taken from compilations of experimental results. In other words, it is a parameter-free model. The starting point is a gene expression (GE) description^4^, where small portions of a tissue define microstates in GE space. In this picture, the normal (homeostatic) and tumor states are seen as distant regions (attractors)^5,6^. On the other hand, the high dimensionality of the GE space, coming from the large number of differentially expressed genes, can be reduced by means of principal component analysis^7–9^. This procedure has been recently applied in Refs.^10,11^ to the analysis of gene expression data for 15 types of cancer from The Cancer Genome Atlas portal^12^, showing very interesting results. In particular, the first principal component axis measures progression to cancer. Based on the results from Refs.^10,11^, especially the case of colon adenocarcinoma (COAD) which is discussed in detail in this paper as a prototype, we aim at building a simplified parameter-free one-variable model for the cancer risk.

## A one-dimensional model for tumorigenesis

As mentioned above, we want to develop a parameter-free one-dimensioinal model for carcinogenesis, which is tested against experimental data from 8 tissues corresponding to the cancer types marked in bold in Table 1. In order to describe in detail the model, we use as example the adenocarcinoma in colon (COAD).

**Table 1:** TCGA abbreviations for the studied cancer types.

Abbreviation	Cancer type
BLCA	Bladder Urothelial Carcinoma
**BRCA**	Breast invasive carcinoma
**COAD**	Colon adenocarcinoma
**ESCA**	Esophageal carcinoma
**HNSC**	Head and and neck squamous cell carcinoma
KIRC	Kidney clear cell carcinoma
KIRP	Kidney papillary cell carcinoma
**LIHC**	Liver hepatocellular carcinoma
**LUAD**	Lung adenocarcinoma
LUSC	Lung squamous cell carcinoma
PRAD	Prostate adenocarcinoma
READ	Rectum adenocarcinoma
STAD	Stomach adenocarcinoma
**THCA**	Thyroid carcinoma
UCEC	Uterine corpus endometrial carcinoma

**Figure 1:**
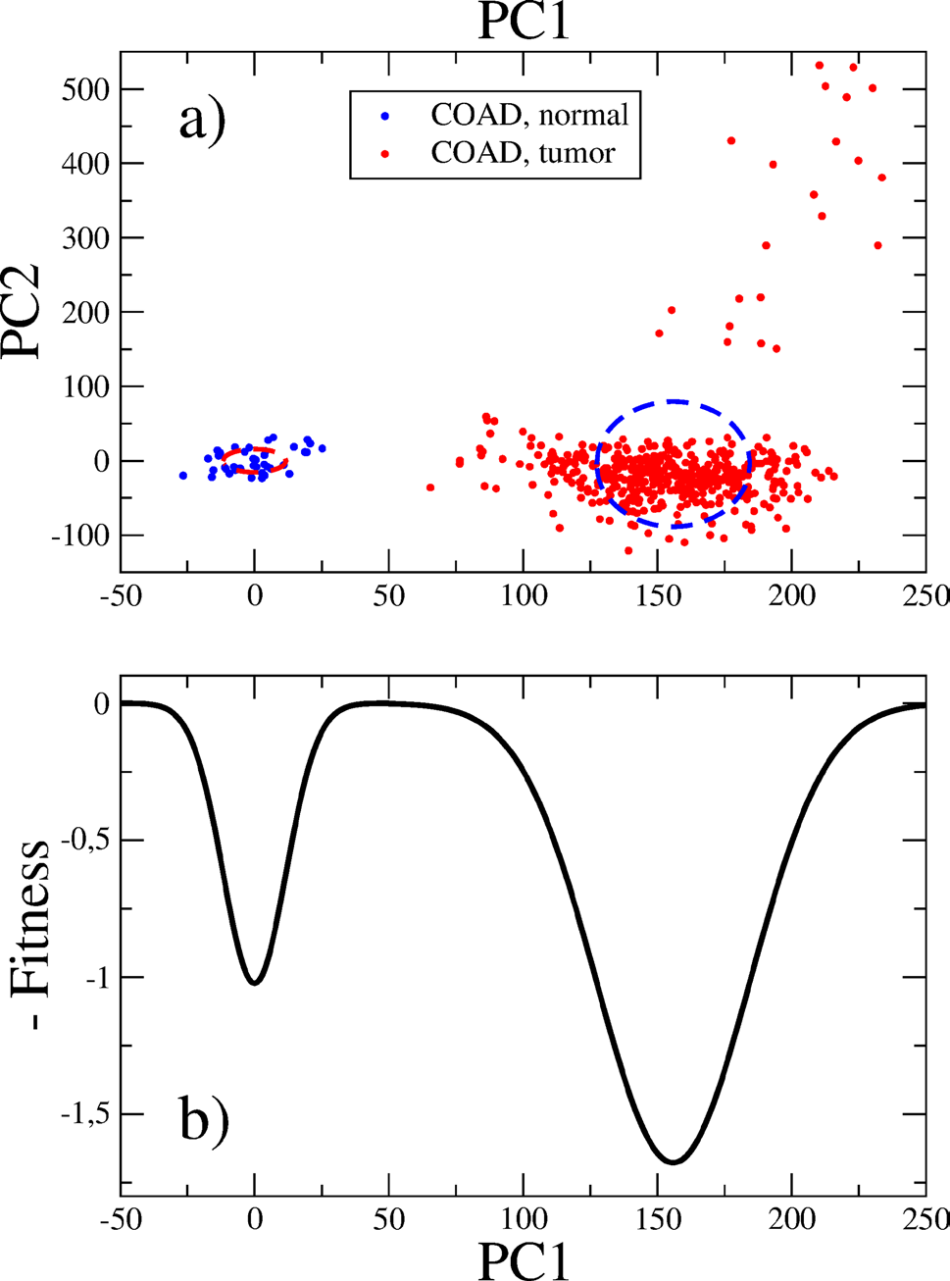
(**a**) PC analysis of the GE data for adenocarcinoma of the colon. Normal (blue circles) and tumor samples (red circles) are shown. Ellipses illustrating the centers and r.m.s. radii of both clouds of points are drawn. (**b**) Schematics of the fitness landscape. The fitness is normalized to the homeostatic value. The tumor region exhibits the deepest well (highest fitness).

We plot in Fig. 1 top panel the results of the principal component analysis methodology^10,11^ applied to GE data for colon adenocarcinoma (COAD). Each point in this figure comes from a biopsy, small samples are taken off from different patients and processed in order to obtain expression values for 60483 genes. For each gene, we define a reference value, $e_{ref}$, by geometric averaging over the normal (healthy) samples. Then, new variables are defined: $\hat{e}=\log _2(e/e_{ref})$^10,11^. The origin of coordinates in this figure is precisely the center of the cloud of normal samples, $\hat{e}=0$. A covariance matrix is defined and diagonalized. The first eigenvectors are used to define new coordinate axes: PC1, PC2, etc. Details may be found in Ref.^11^. We shall only stress that the first axis, PC1, which accounts for 51 % of the total data variance, is the cancer axis, which allows the discrimination between normal and tumor states. The position along PC1 is then the variable to be used in our model.

Let $\mathbf{v}_\mathbf{1}$ be the eigenvector of the covariance matrix along PC1, and ${\hat{\mathbf{e}}}$ the expression vector corresponding to a given sample. Then, $x_1={\hat{\mathbf{e}}}\cdot \mathbf{v}_\mathbf{1}$ is the position along PC1 of the sample. Normal samples define a region around the origin with r.m.s. radius $R_n=11.71$. On the other hand, the cloud of tumor samples is centered at $\bar{x}_1=155.89$, and its r.m.s. radius is $R_t=28.53$^10^.

Recall the interpretation of points in Fig. 1 top panel. Each point comes from a small sample, the GE data obtained from it contains the contribution of many cells and the complex signaling system regulating their interactions. One may speak of a tissue microstate. On the other hand, points come from different patients, each carrying a particular genetic load. The fact that the points are grouped in definite regions means that these regions are indeed attractors in GE space.

We want to describe the genesis of a tumor, that is the time evolution of a portion or sample of a tissue that starts in the normal region and progress towards the tumor zone. We have already defined a single coordinate describing this progression: $x_1$. In order to proceed further with the model, we shall clarify why and how this progression takes place.

The coordinate $x_1$ describing the tissue microstate starts at a point near the origin and realizes random oscillations in the normal zone. The cause for such random displacements is discussed in the next sections. The motion is confined to the normal region for a long time because this zone is a local maximum of fitness^10,13^. We have schematically represented in Fig. 1 bottom panel the fitness distribution along the PC1 axis. The *y* axis of this figure is the fitness with a minus sign, thus that the normal and tumor zones are local maxima of fitness. In the figure, the fitness is estimated from the histogram of samples along PC1. We have computed in Ref.^13^ the number of available microstates in each zone, showing that this number is much greater for tumors than for normal states. In other words, the volume of the basin of attraction is much greater in the tumor than in the normal region. In addition, as a consequence of breaking the restrictions imposed by homeostasis, the mitotic rate of tumor stem cells is usually greater than that of normal somatic stem cells^14^. The conclusion is that the tumor minimum should be the deepest in Fig. 1, the one with highest fitness. Our drawing for the fitness distribution is a sketch built from the available data, however we are convinced that it is a qualitatively correct representation of the actual fitness distribution.

The intermediate region, $R_n<x_1<\bar{x}_1-R_t$, holds a low-fitness barrier^10,13^, as shown in Fig. 1 bottom panel, which prevents the spontaneous transitions from the normal to the tumor region. The relative scarcity of samples in this region evidences the existence of the barrier.

A tissue microstate realizes random displacements within the normal region. Only when the barrier is surpassed and the microstate leaves the normal basin of attraction it is driven towards the tumor attractor. The transition is seen as discontinuous^10^.

A precise description of the transition requires the detailed knowledge of the fitness landscape and the causes of the random fluctuations. However, in order to estimate the risk of cancer in a tissue we may proceed in a simpler way and compute the probability for the $x_1$ variable describing the microstate to transit from the normal to the tumor region. The minimal walk length is $\bar{x}_1-R_n-R_t$. This is the goal we are aimed at in the present paper.

The starting point in our model is a large set of samples or microstates located near the origin of Fig. 1. They represent small portions of the healthy tissue. We may think of colon crypts in the studied example. The mean number of crypts in a healthy individual is estimated in Ref.^15^ as $1.5\times 10^7$. We shall follow the random oscillations in GE space of each of these crypts.

With regard to the time variable, it is natural to follow the renewal cycle of somatic stem cells, guaranteeing crypt homeostasis. In the studied example, the renewal rate is 73 per year^16^. Thus, we shall measure time in terms of somatic stem cell generations. $t=0$ may refer to conception or to the moment at which the first colon stem cell appears. On the other hand, $t_0=\log _2 N_{sc}$, where $N_{sc}$ is the number of stem cells in the tissue, is the moment at which the tissue is formed. In colon, $N_{sc}\approx 2\times 10^8$, and $t_0\approx 27$. This is our starting point.

## Small random displacements in GE space

Any variation in the expression of genes is a displacement in GE space. We conceptualize two kinds of GE variations: small displacements and large rearrangements. Naively, one may relate small displacements to variations in the expression of one or a few genes, whereas large GE rearrangements are coordinated variations of the expressions of many genes.

Small variations of GE levels spontaneously occurs and may have different origins. First, somatic mutations in the human genome are known to occur at a rate of 8 per cell generation^17^. Second, there is also a rate of accumulation of epigenetic (mainly methylation and phosphorylation) events modifying the normal expression levels^18^. Both processes could be boosted by inherited mutations^19,20^ or external carcinogens^21^.

We may thus write for the $x_1$ coordinate, characterizing the microstate of a crypt at time $t=n+1$, the following equation:

1 $$\begin{aligned} x_1^{(n+1)}=x_1^{(n)}+\delta x_1, \end{aligned}$$

where

2 $$\begin{aligned} \delta x_1=\mathbf{v}_\mathbf{1}\cdot \delta {\hat{\mathbf{e}}}=\sum _{i}v_{1i}\,\delta \hat{e}_i, \end{aligned}$$

and $\delta \hat{e}_i$ corresponds to a random variation of the expression of the *i*-th gene. Eq. (1) describes a Markov chain of events^22^. On the other hand, Eq. (2) shows that fluctuations in the expression levels are filtered by the $\mathbf{v_1}$ vector.

**Figure 2:**
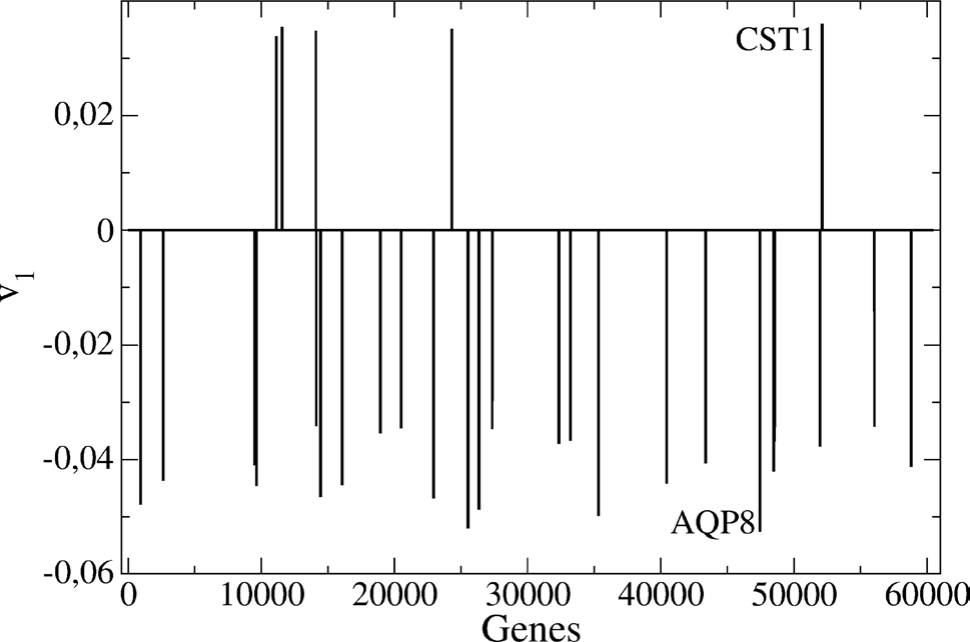
The 30 genes with most significant contributions to the $\mathbf{v_1}$ vector in COAD. The x axis is the sequence number of a given gene in the TCGA data. CST1 is highlighted among the over-expressed and AQP8 among the silenced genes.

In Fig. 2 we draw the 30 genes with the greatest contributions to $\mathbf{v_1}$ in COAD^11^. Positive, $v_{1i}>0$, and negative, $v_{1i}<0$, amplitudes correspond respectively to over- and under-expressed (silenced) genes in the tumor progression. We have distinguished the genes CST1 and AQP8. The former is a known marker of colon cancer^23^, whereas the latter plays a significant role in colon homeostasis^24^ and should be silenced in tumors.

The maximum value of $|v_{1i}|$ defines a scale, *D*, for the fluctuations of $x_1$. In COAD, it coincides with the modulus of the $v_{1i}$ related to the AQP8 gene. In order to get a simple estimate for the cancer risk, we may adopt the following model for the fluctuations: $\delta x_1=D\,r$, where *r* is a uniformly distributed random number in (-1,1). This model may result from an independent variation hypothesis, i.e. random amplitudes and signs in the individual gene variations $\delta \hat{e}_i$, so that most of them cancel out. In this way, Eq. (1) for the small displacements in GE space describes a 1D Brownian or Poisson process^25^.

We may use the well known fact that in a Brownian process, the final amplitudes at a given time are normally distributed, i.e. the probability density is given by:

3 $$\begin{aligned} p(x)=\sqrt{a/\pi }\, e^{-a (x-x_0)^2}, \end{aligned}$$

where $a=2/(D^2 t)$. We shall evaluate the probability for a trajectory starting in the normal zone to reach the tumor zone. Above, we pointed out that the minimal walk length is $R=\bar{x}_1-R_n-R_t$. Thus, an estimate for the risk may be obtained from:

4 $$\begin{aligned} \int _{R}^{\infty } p(x) \mathrm{d}x=\text {Erfc}(\sqrt{a R^2}), \end{aligned}$$

where $\text {Erfc}(z)$ is the complementary error function. The argument of this function is $z=\sqrt{a R^2}=\sqrt{2/t}\,R/D$, in principle a large number. Then, we may use the asymptotic behavior $\text {Erfc}(z)\approx \exp (-z^2)/(\sqrt{\pi }z)$ for large *z*. The risk of cancer in COAD is obtained by multiplying the escape probability for a single crypt by the number of crypts, or by the number of stem cells, which is proportional to it:

5 $$\begin{aligned} risk \sim N_{sc}\, \frac{D\sqrt{t}}{R}\,e^{-2(R/(D\sqrt{t}))^2}, \end{aligned}$$

or

6 $$\begin{aligned} ln (risk/N_{sc}) = const + ln(D\sqrt{t}/R)-2(D\sqrt{t}/R)^{-2}. \end{aligned}$$

This expression is general enough to be applied to other tissues, besides colon. The constant in Eq. (6) may account for other effects as, for example, the role of the immune system. Microregions escaping the normal region and forming a prototumor could be the subject of an attack by the immune system in the very early stages^26^. By definition, the constant is less than zero because the overall constant in Eq. (5) is less than one.

In Table 2 we compile a set of parameters for a group of tumors. The geometry of the normal and tumor regions, i.e. the parameters $\bar{x}_1$, $R_n$ and $R_t$ come from Ref.^10^. The *D* value is estimated as the maximum of $|v_{1i}|$^11^. On the other hand, the number of tissue stem cells, $N_{sc}$, the stem cell turnover rate, $m_{sc}$, and the lifetime risk of cancer (when available) are borrowed from Refs.^27,28^. The reported values of risk represent averages over 380 cancer registries from different cities and countries around the world^28^.

**Table 2:** A set of parameters compiled for a group of tumors.

**Tissues**	$\bar{x}_1$	$R_n$	$R_t$	*R*	*D*	$N_{sc}$	$m_{sc}$ (1/yr)	$\langle \hbox {risk}\rangle$	$\langle \hbox {dev}\rangle$
BLCA	140.61	57.53	34.68	48.40	0.0512	.	.	.	.
**BRCA**	137.37	20.97	31.66	84.74	0.0450	$8.7\times 10^9$	4.3	0.09228	0.03427
**COAD**	155.89	11.71	28.53	115.65	0.0526	$2\times 10^8$	73	0.04264	0.01504
**ESCA**	138.70	64.28	35.79	38.63	0.0710	$6.65\times 10^6$	33.18	0.00412	0.01378
**HNSC**	123.50	27.74	23.54	72.22	0.0549	$1.85\times 10^7$	21.15	0.01527	0.00578
KIRC	171.81	28.70	36.01	107.09	0.0679	.	.	.	.
KIRP	163.42	19.90	27.78	115.74	0.0768	.	.	.	.
**LIHC**	134.67	20.48	45.23	68.96	0.0461	$3.01\times 10^9$	0.9125	0.00397	0.00310
**LUAD**	145.33	13.52	32.06	99.75	0.0581	$1.22\times 10^9$	0.07	0.01610	0.00847
LUSC	194.49	11.62	36.65	146.22	0.0522	.	.	.	.
PRAD	91.33	31.31	32.17	27.85	0.0523	$2.1\times 10^8$	3	0.13712	0.07730
READ	168.05	22.90	28.81	116.34	0.0521	.	.	.	.
STAD	136.97	27.14	43.24	66.59	0.0455	.	.	.	.
**THCA**	112.55	20.02	39.85	52.67	0.0532	$8.25\times 10^7$	0.087	0.00649	0.00442
UCEC	171.38	38.24	22.14	111.00	0.0439	.	.	.	.

The geometry of the normal and tumor regions, i.e. the parameters $\bar{x}_1$, $R_n$ and $R_t$ come from Ref.^10^. The minimal distance between both regions is $R=\bar{x}_1-R_n-R_t$. The *D* value is estimated as the maximum of $|v_{1i}|$^11^. On the other hand, the number of tissue stem cells, $N_{sc}$ and the stem cell turnover rate, $m_{sc}$, are borrowed from Refs.^27,28^. The lifetime risk of cancer and its deviation (when available) is computed from Ref.^28^ as the mean value and the standard deviation of the cumulative risk at a maximum age of 80 years. Bold marked tissues correspond to cancer types for which all the data is available.

We may test Eq. (6) for the risk of cancer in a tissue resulting from small random variations of GE levels by using the data included in Table 2. A plot of the l.h.s. vs the r.h.s. of Eq. (6) should lead to a straight line with a slope near one and a constant less than zero. Notice that the life expectancy in Ref.^27^ is assumed to be 80 years. Thus, *t* is obtained by multiplying the stem cell rate, $m_{sc}$, by 80 years.

**Figure 3:**
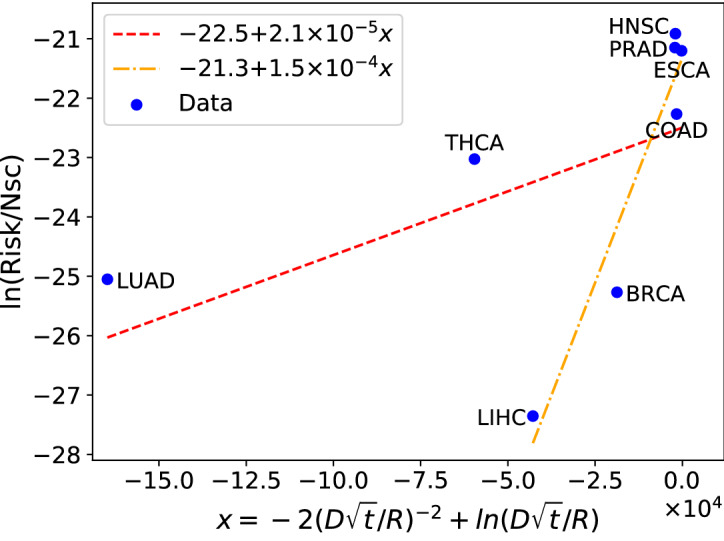
A test of how Eq. (6) describes the risk of cancer in 8 tissues. Data from Table 2 is used to this end. A very small slope is obtained in both, the full fit and a fit without LUAD and THCA, thus small amplitude fluctuations in gene expression space may not account for the risk of cancer in these tissues.

The results of that test are shown in Fig. 3. We get a nearly flat curve (slope = $2.1\times 10^{-5}$, or $1.5\times 10^{-4}$ if we leave LUAD and THCA out of the fit), indicating that the proposed dependence of the risk on the parameters is not correct. Thus, the observed risk of cancer can not be explained by random variations of small amplitude in GE values. In the next section, we shall consider large GE rearrangements, or equivalently large jumps in GE space.

Let us stress that we use an expression like $t=m_{sc}\times age$ in a very broad age interval. It is well known that $m_{sc}$ experiences a significant decrease as a result of aging^29,30^. However, also as a consequence of aging there is an accumulation of epigenetic events and DNA damages leading to a reduction of fitness and a displacement towards the low-fitness zone. Thus, aging acts in the same direction as the low amplitude fluctuations of GE values.

## Large (Levy) jumps in GE space

Besides small random displacements, related to quasi independent variations in the GE values, there is also the possibility of large jumps in GE space. The origin of such large motions could be diverse.

First, there are large scale mutations, involving DNA rearrangements and simultaneously modifying the expression of many genes. An example, known to play an important role in cancer, is that of aneuploidies^31^.

Second, large jumps in GE space could be related to coordinated variations in a group of genes. Indeed, GE values are known to be regulated by GE networks^32^. The global states of these networks define attractors^5,6^. Variations in genes playing a decisive role in the network, or accumulation of variations in many genes, may cause a transition from one of these global states to another one.

Third, there is also the possibility of a programmed chain of GE variations leading to cancer, triggered by yet unknown causes, which is the basic hypothesis in the atavistic theory of cancer^33^.

For the large GE variations, we shall specify their rate of occurrence, $\mu$, and the probability distribution for their amplitudes, $\pi (\Delta x_1)$. It is very plausible to assume that $\pi$ is of Pareto^34^ or Levy^35^ kind, with a power-like tail. Indeed, the Pareto character of GE distribution functions was demonstrated in Ref. ^36^ (see also ^10^). The Levy character of the length distribution functions in mutations was shown in^37^.

Thus, our assumption is that displacements in GE space are a kind of Levy flights. Small variations allow the exploration of the fitness landscape at lower scales, whereas sporadic large jumps allow to find global maxima. Besides mutations^37^, Levy flights are known to take place in many other biological processes, for example foraging^38^.

For lage $|\Delta x_1|$, the tail of $\pi$ is described by a Pareto exponent $\nu$:

7 $$\begin{aligned} \pi (\Delta x_1)\sim 1/|\Delta x_1|^\nu . \end{aligned}$$

The probability of a large jump reaching the tumor region is thus proportional to

8 $$\begin{aligned} D \,(\mu t)\, \int _{R}^{\infty } \mathrm{d}x/x^\nu , \end{aligned}$$

and the risk of cancer in a tissue:

9 $$\begin{aligned} risk\sim N_{sc}D\, \mu \, (t_0+m_{sc}\times age)/R^{\nu -1}, \end{aligned}$$

where we assume $\nu >1$. Below, we use $\nu =2$ in order to get an estimate of the risk.

Let us examine Eq. (9) in more details. First, Eq. (8) assumes that *R* is in the tail of the distribution function. This is justified if we compare *R* with the scale *D*, that in the case of COAD take the values of $R = 115.65$ and $D = 0.0526$ respectively. Second, no more than one hit or large jump is assumed to occur in the evolution of each microstate. In other words, the probability $\mu t$ is less than one and large jumps are thought to be rare. Third, we should consider the possibility of large GE variations in the development period, that is why we included $t_0=\log _2(N_{sc})$ in the formula. This is particularly important in tissues with slow renewal rates but large number of stem cells. For example, in lung $t_0\approx 30$, but $m_{sc}\times 80$ years is only 5.6. Fourth, the rate of large jumps, $\mu$, is unknown. However, if we assume roughly the same value for all tissues, then it can be absorbed in the overall constant entering Eq. (9). The Pareto exponent is also unknown. Notice that in the GE distribution functions of COAD the exponents take values between 1.6 and 2.0^10^. The value we use for estimates, $\nu = 2$, is motivated by this result.

**Figure 4:**
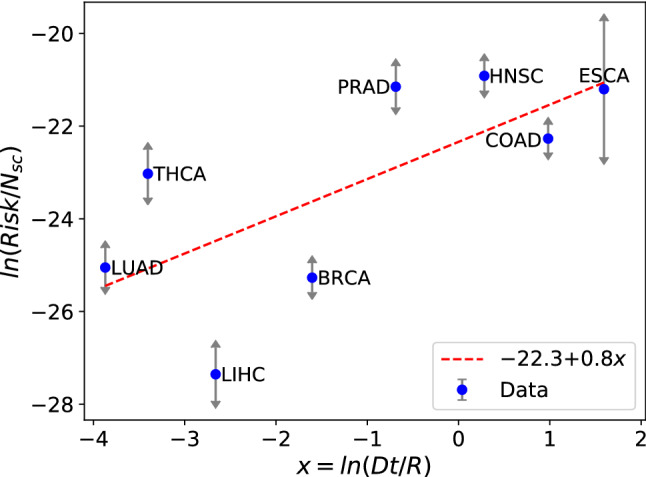
Testing the ability of Eq. (10) to describe the cancer risk in 8 tissues. The error bars were estimated by means of the last column of Table 2. The slope of the linear fit is near one, as expected. 72 % of the data dispersion is explained by the linear dependence (p-value = 0.04).

Finally, we get the following expression for the risk, which may be tested against the data in Table 2:

10 $$\begin{aligned} ln (risk/N_{sc})=const + ln (D t/R), \end{aligned}$$

where $t=t_0+m_{sc}\times age$. The constant should be negative according to our hypothesis of $\mu$ small. The results of the test with an average life expectancy of 80 years are shown in Fig. 4.

The observed behavior is consistent with a linear dependence with slope near one. Indeed, we obtain a slope equal to 0.82. The Pearson correlation coefficient is 0.85, indicating that 72% of the dispersion of points may be explained by a linear dependence. The p-value is equal to 0.04. The small error bars suggest that the main reason for the unexplained dispersion of points could be the assumption that the rate of large jumps, $\mu$, is roughly the same for all of the tumors. A tissue specific $\mu$ variable would account for the dispersion.

In conclusion, we get the following simple expression for the risk of cancer per stem cell in a tissue, coming from large jumps in GE space:

11 $$\begin{aligned} \frac{risk}{N_{sc}}=\mu ' \frac{D}{R} t, \end{aligned}$$

where we included an effective rate, $\mu '$. Genetic, viral or external carcinogenic factors may increase $\mu '$, whereas the action of the immune system in the tissue may modify $\mu '$ in any direction. In the next section, we qualitatively analyze a larger set of tumors by using Eq. (11).

## Qualitative analysis of the data on cancer risk in different tissues

We use the extremely simple expression for the cancer risk in a tissue, offered by Eq. (11), in order to re-examine the data presented in paper ^27^. The idea is to rewrite the coefficients in front of the r.h.s of Eq. (11), as $ERS \times a_{ref}$, where $a_{ref}=2\times 10^{-14}$ is a reference value and ERS is an extra risk score. Eq. (11) is thus rewritten as:

12 $$\begin{aligned} \frac{risk}{N_{sc}}=ERS\, a_{ref}\, (t_0+m_{sc} age). \end{aligned}$$

**Figure 5:**
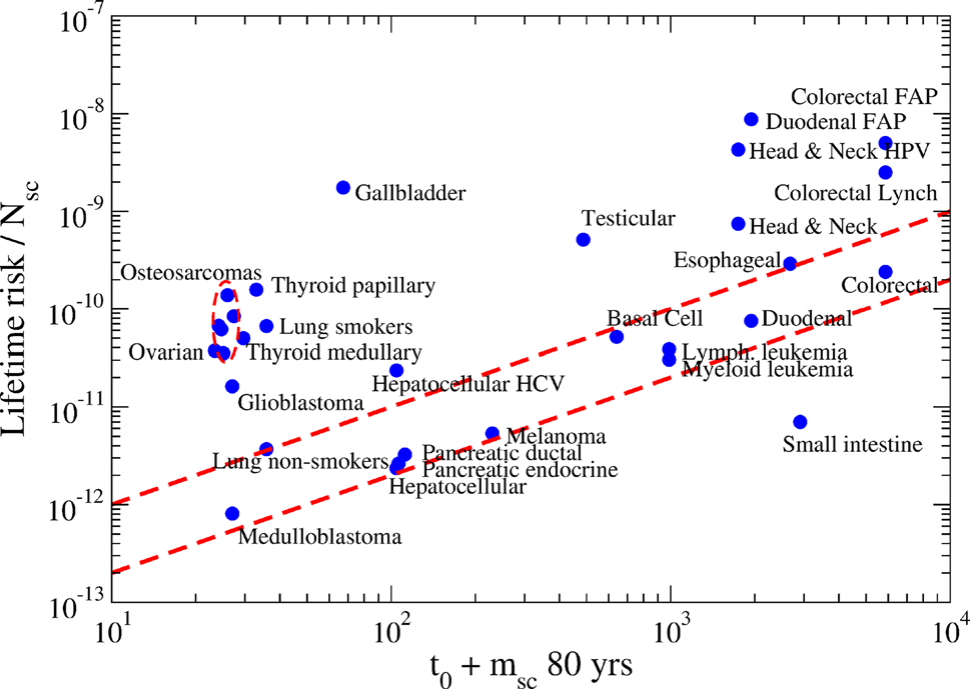
Lifetime risk of cancer per stem cell in a tissue vs the number of stem cell generations. The analysis is based on Eq. (12). The band delimited by the red dashed lines contains the group of tissues qualified as normal. See detailed explanations in the main text.

This expression provides a simple explanation for the intuitive claim in Ref.^27^ that the risk is related to the number of stem cell replications. The results are shown in Fig. 5 and Table 3. We should try to understand the observed values of ERS in terms of the tissue characteristics. In order to facilitate the analysis, the studied tumors are separated in groups.

**Table 3:** The Extra Risk Score (ERS) index of Eq. (12) for cancer in different tissues.

Cancer type	ERS
**Group I. Normal**	
Hepatocellular C	1.13
Melanoma	1.16
Pancreatic endocrine C	1.23
Pancreatic ductal AC	1.45
Medulloblastoma	1.49
Myeloid leukemia	1.54
Duodenal AC	1.93
Lymphocytic leukemia	1.95
Colorectal AC	2.04
Basal Cell C	4.02
Lung AC (non-smokers)	5.15
Esophageal SCC	5.44
**Group II. Viral and Genetic**	
Hepatocellular C with HCV	11.29
Colorectal AC with Lynch	21.30
Colorectal AC with FAP	42.61
Head and Neck SCC with HPV	122.96
Duodenal AC with FAP	225.29
**Group III. Immune**	
Small intestinal AC	0.12
Glioblastoma	30.03
Testicular germinal cell	52.78
Osteosarcomas Head	70.03
Ovarian germinal cell	79.87
Thyroid medullary C	84.22
Osteosarcomas Arms	124.72
Osteosarcomas Pelvis	138.09
Osteosarcomas	153.05
Thyroid papillary and follicular C	239.78
Osteosarcomas Legs	266.49
Gallbladder non papillary AC	1299.58
**Group IV. Abnormal**	
Head and Neck SCC	21.38
Lung AC (smokers)	92.77

Group I includes 12 tumors (10 tissues), located in a band delimited by red dashed lines in Fig. 5, and coefficients $1<ERS<6$. In the lack of a better name, it is called the normal group. In this set, random fluctuations in GE space seem to play the main role in the genesis of cancer, as originally claimed in Ref.^27^. Notice that this group is conformed by very different tissues, from the medulloblastoma to the colorectal adenocarcinoma.

Group II, with five points in the figure, include cases in which genetic or viral causes enhance the rate $\mu '$. The ERS index exhibits very high values in this set.

The abnormal values of ERS for the 7 tissues (12 points) contained in Group III could have an immunological origin. Indeed, our body uses physical barriers, humors and immune cells in order to protect the tissues against infections caused by pathogens, which are the most common attacks. The combined effects of these factors guarantees immunity. In tissues where one factor is predominant, the others could be somehow depressed. On the other hand, the protection against tumors, which come form inside, that is originate in tissue cells, is mainly the responsibility of immune cells. In other words, in tissues where the role of immune cells is depressed at the expense of increasing barriers or other components, the relative cancer risk, and correspondingly the ERS factor, is increased.

Barriers are known to play a basic role in the protection of germinal cells^39^ and the brain^40^ against infections. The cellular component of immunity in these tissues is, in some way, depressed with the purpose of avoiding inflammation events. The relatively high values of ERS could be explained in this way.

By contrast, the inclusion of the Medulloblastoma in the normal group is probably related to regional differences in blood-brain barrier permeability^41^.

With regard to bones, it is known that immunity relies strongly on defensins^42^, possibly with a depressed role of immune cells. On the other hand, the thyroid is known to have a close cross-talk with the immune system^43^. It’s dysregulation is the cause of immune disorders. One may speculate that a low cellular response is needed in order to prevent dysregulation of the thyroid.

The extreme case in this group is gallbladder non-papillary adenocarcinoma, with an index $ERS=1300$, the understanding of which is a real challenge. However, one can speculate that the cellular response is also depressed in the gallbladder, because of the strong microbicide character of the bile^44^.

On the other hand, the relatively low value of ERS for the small intestine adenocarcinoma (eight times lower than the reference) can not have other explanation than overprotection by the cellular component of the immune system. Indeed, the small intestine is a possible entrance door for the microbiota living in the colon, and as such it requires special protection. The mean value of microbes/gm experiences a jump from $10^4$ to $10^{11}$ as we cross from the ileum to the cecum^45^. Barriers can not be reinforced because of the reduced dimensions. Thus, perhaps the Paneth cells^46^, Peyer’s patches^47^, and other structures concentrated in the distal ileum are the responsible for this additional protection.

Finally, there is a group of 2 tissues exhibiting abnormally high values of the ERS index, presumably related to external factors. One example is lung adenocarcinoma, for which the concurrence of radioactive Radon and smoking produces a 90-fold increase of the slope.

## Concluding remarks

In the present paper, the time evolution of microstates representing small portions of a tissue are described as Levy flights in gene expression space. The small amplitude Brownian component is characterized by a radius $D \sqrt{t}$, much less than the distance between the normal and tumor regions, $R=\bar{x}_1-R_n-R_t$. Only sporadic large jumps, of Levy nature, allow the microstate to reach the cancer basin of attraction, and thus explain the risk of cancer in a tissue.

Although it is understood that aging induces a motion in the direction of the low-fitness region, it was not explicitly included in our model. Work along this direction is necessary.

The resulting formula for the risk of cancer in a tissue was quantitatively tested against the observed data in 8 tissues, and applied to the qualitative analysis of a risk of cancer in a larger set of tissues. The most important conclusion, in our opinion, is a possible connection between the risk and the way the tissue is protected against infections. The blood-brain barrier in the cerebrum, for example, preventing the entrance of pathogens, is also the reason for the relatively low rate of elimination of prototumors, and thus large risk per stem cell in this organ. The low risk per stem cell in the small intestine, on the other hand, is understood as a reinforcement of the cellular component of immunity.

## Data Availability

The information about the data we used, the procedures and results are integrated in a public repository that is part of the project “Processing and Analyzing Mutations and Gene Expression Data in Different Systems”: https://github.com/DarioALeonValido/evolp. In particular, the data we use from Refs.^27,28^ is replicated in ../evolp/databases_external/Cancer_Risk/. The principal component analysis (PCA) on gene expression data downloaded from The Cancer Genome Atlas is located in ../evolp/databases_generated/TCGA_pca/. We include a specific python script for this work that can be found in the folder ./evolp/Levy_cancer/. The script produces two data files with the information contained in Tables 2 and 3, and two figures similar to the ones presented in the paper.
